# Ubiquilin-2 drives NF-κB activity and cytosolic TDP-43 aggregation in neuronal cells

**DOI:** 10.1186/s13041-015-0162-6

**Published:** 2015-10-31

**Authors:** Vincent Picher-Martel, Kallol Dutta, Daniel Phaneuf, Gen Sobue, Jean-Pierre Julien

**Affiliations:** Research Centre of Institut Universitaire en Santé Mentale de Québec, Laval University, 2601 Chemin de la Canardière, Québec, QC G1J 2G3 Canada; Department of Psychiatry and Neuroscience, Laval University, 2601 Chemin de la Canardière, Québec, QC G1J 2G3 Canada; Department of Neurology, Nagoya University Graduate School of Medicine, 65 Tsurumai-cho Showa-ku, Nagoya, 466-8550 Japan

**Keywords:** Amyotrophic lateral sclerosis (ALS), Ubiquilin-2 (UBQLN2), TAR DNA-binding protein 43 (TDP-43), NF-κB p65, p38 MAPK, ER-stress, Neuronal death, Withaferin A (WA)

## Abstract

**Background:**

Mutations in the gene encoding Ubiquilin-2 (UBQLN2) are linked to amyotrophic lateral sclerosis (ALS) and frontotemporal dementia (FTD). UBQLN2 plays a central role in ubiquitin proteasome system (UPS) and UBQLN2 mutants can form cytoplasmic aggregates in vitro and in vivo.

**Results:**

Here, we report that overexpression of WT or mutant UBQLN2 species enhanced nuclear factor κB (NF-κB) activation in Neuro2A cells. The inhibition of NF-κB stress-mediated activation with SB203580, a p38 MAPK inhibitor, demonstrated a role for MAPK in NF-κB activation by UBQLN2 species. Live cell imaging and microscopy showed that UBQLN2 aggregates are dynamic structures that promote cytoplasmic accumulation of TAR DNA-binding protein (TDP-43), a major component of ALS inclusion bodies. Furthermore, up-regulation of UBQLN2 species in neurons caused an ER-stress response and increased their vulnerability to death by toxic mediator TNF-α. Withaferin A, a known NF-κB inhibitor, reduced mortality of Neuro2A cells overexpressing UBQLN2 species.

**Conclusions:**

These results suggest that UBQLN2 dysregulation in neurons can drive NF-κB activation and cytosolic TDP-43 aggregation, supporting the concept of pathway convergence in ALS pathogenesis. These Ubiquilin-2 pathogenic pathways might represent suitable therapeutic targets for future ALS treatment.

**Electronic supplementary material:**

The online version of this article (doi:10.1186/s13041-015-0162-6) contains supplementary material, which is available to authorized users.

## Background

Amyotrophic lateral sclerosis (ALS) is the most common adult-onset motor neuron disorder. It is characterized by progressive degeneration of upper and lower motor neurons leading to paralysis and, unfortunately, to patient’s death within 2 to 5 years. Nearly 10 % of ALS cases are familial and 90 % are sporadic. Expanded hexanucleotide repeats in C9orf72 account for approximately 30 % of familial cases, mutations in superoxide dismutase 1 (SOD1) for 20 % whereas other genes like TAR DNA-binding protein (TDP-43), fused in sarcoma (FUS), p62/SQSTM1 and Ubiquilin-2 (UBQLN2) account for less than 10 % [1]. The main pathogenic mechanisms of ALS are still a mystery. Numerous cellular dysfunctions have been linked to ALS physiopathology including oxidative stress, protein inclusions, inflammatory processes, RNA processing and endoplasmic reticulum stress (ER-stress) [2].

Ubiquilin-2 acts as an important player in the ubiquitin proteasome system (UPS) by connecting the UPS and ubiquitinated proteins. It is also implicated in autophagy, cell cycle progression and cell signaling. UBQLN2 possesses an N-terminal ubiquitin-like domain, a C-terminal ubiquitin-associated domain and a PXX domain essential for protein-protein interaction [3]. Originally, five X-linked mutations in UBQLN2 gene have been discovered in ALS/FTD familial cases [4]. All these mutations are located in the PXX domain and one of the most frequent is P497H. Patients with mutant UBQLN2^P497H^ develop cytoplasmic inclusions positive for major proteins implicated in ALS such as TDP-43, ubiquitin, FUS and p62. Furthermore, ALS/FTD patients without UBQLN2 mutation also express UBQLN2 positive inclusions, supporting an important role of this protein in ALS physiopathology [4]. More than ten UBQLN2 mutations have been currently described in ALS, not only in the PXX domain [5–8]. UBQLN2 is also implicated in other neurological disorders such as FTD [4], Alzheimer’s disease [9] and Huntington’s disease [10].

Nuclear Factor kappa-B (NF-κB) is a transcription factor implicated in inflammation. NF-κB is formed by members of Rel/NF-κB family such as p50, p52, p65 (RelA), RelB or c-Rel in homo or heterodimeric complexes. The complex composed of p65 and p50 has been the most characterized. A wide variety of extracellular signals lead to NF-κB activation, including cytokines, infectious agents or oxidants. Almost all signals that trigger the NF-κB signaling pathway converge on activation of a molecular complex that contains a serine residue-specific IκB kinase (IKK). In the classical (canonical) NF-κB pathway, activation of the IKK complex leads to phosphorylation mediated by IKKβ of IκB-α, which is subsequently targeted for intracellular ubiquitination and degradation by the proteasome complex. This releases p65 NF-κB from IκB-α inhibitor and the phosphorylated p65 form is then transported to nucleus where it binds to specific response elements (RE) affecting transcription of various genes involved in a diversity of biological processes such as immunity, inflammatory, stress response and development [11]. NF-κB has an emerging role in ALS or other neurological disorders. NF-κB activity is increased in human neuroblastoma cells expressing mutant SOD1^G93A^ [12] and it is up-regulated in motor neurons of sporadic ALS cases [13]. Our group reported previously that TDP-43 interacts with NF-κB and that NF-κB mRNA levels are abnormally up-regulated in the spinal cord of ALS patients [14]. Furthermore, NF-κB inhibition by administration of Withaferin A, a known NF-κB inhibitor, reduced ALS disease symptoms in a TDP-43 transgenic mouse model [14] and extended lifespan of mutant SOD1 ALS mice [15]. Longevity of mutant SOD1 mice was also increased by microglia-specific inhibition of NF-κB pathway [16]. These data suggest a central role for the NF-κB pathway in ALS pathogenesis.

Here, we used a NF-κB-luciferase reporter assay to examine the effect of UBQLN2 overexpression on NF-κB activity. We have determined that up-regulation of UBQLN2 enhances NF-κB activation in Neuro2A cells. We also used small interference RNA (siRNA) against UBQLN2 to prevent NF-κB activation by UBQLN2. Treatment of transfected cells with different MAPK inhibitors suggested that NF-κB activation by UBQLN2 species resulted from p38 MAPK activation. Moreover, we found that up-regulation of UBQLN2 protein species causes aggregation and cytoplasmic accumulation of TDP-43. Evidence is presented that UBQLN2 inclusions are dynamic structures which can increase in size over time. Finally, we report that UBQLN2 upregulation enhances ER-stress response and NF-κB-mediated neuronal vulnerability to death caused by exposure to toxic mediator TNF-α.

## Results

### UBQLN2 up-regulation induces NF-κB activation

**Fig. 1:**
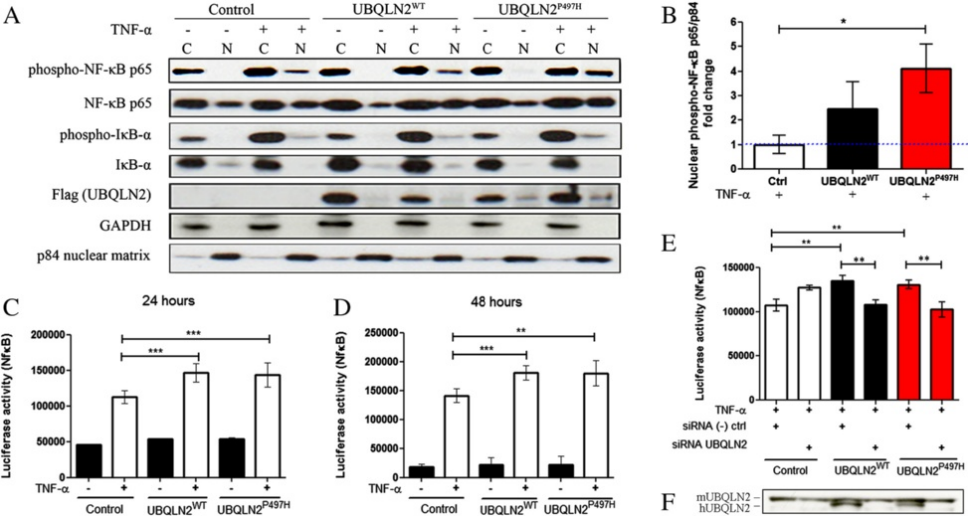
NF-κB activation induced by hUBQLN2 is suppressed with hUBQLN2 siRNA. Neuro2A cells were transfected with control plasmid, pCMV-hUBQLN2^WT^ or pCMV-hUBQLN2^P497H^. Cells were treated or not with TNF-α 20 ng/ml for 4 h before luciferase assay or before collecting the cells. **a** Cytoplasmic (C) and nuclear (N) extraction was realized 48 h after transfection. Antibodies were use according to Materials and Methods. **b** Immunoblot quantification of nuclear phospho-NF-κB p65 vs p84 nuclear matrix as compared to nuclear levels in control cells (*p* = 0.0426, *n* = 3). **c** Neuro2A cells were stably transfected with pluc2p-NFκB-RE plasmid and then transfected with control plasmid, pCMV-hUBQLN2^WT^ or pCMV-hUBQLN2^P497H^. Luciferase activity (NF-κB) was measured at 24 h (*s* < 0.0001 for hUBQLN2^WT^ and hUBQLN2^P497H^, *n* = 6,) and (**d**) at 48 h (*p* = 0.0004 for hUBQLN2^WT^ and *p* = 0.0061 for hUBQLN2^P497H^, *n* = 3). **e** Cells were co-transfected with control plasmid, pCMV-hUBQLN2^WT^ or pCMV-hUBQLN2^P497H^ and with hUBQLN2 siRNA or scrambled siRNA. Luciferase activity was measured at 48 h after transfection (*p* < 0.01, *n* = 5). **f** Immunoblot of UBQLN2 proteins. Upper band corresponds to mouse UBQLN2 (endogenous) and lower band to human UBQLN2 (transfected). Immunoblot was showing the reduced levels of only human UBQLN2 after co-transfection with siRNA

We transfected Neuro2A cells with pCMV-hUBQLN2^WT^ and mutant pCMV-hUBQLN2^P497H^ plasmids to determine the effects of overexpressing hUBQLN2 species on activation of NF-κB signaling. TNF-α treatment was used to trigger NF-κB activation and to mimic neuro-inflammatory condition in ALS disease [17]. We detected a NF-κB hyper-activation in Neuro2A cells overexpressing hUBQLN2 species. Figure 1a shows a Western blot analysis of nuclear and cytoplasmic protein extracts at 48 hours after transfection. We prepared nuclear and cytosolic fractions to assess the NF-κB distribution in cells. According to our results, the distribution and the phosphorylation of NF-κB p65 in Neuro2A cells is required to precisely measure NF-κB activity. As compared to control cells, we observed a slight increase of nuclear phospho-p65 in hUBQLN2^WT^-transfected cells and a higher increase of nuclear phospho-p65 in cells transfected with hUBQLN2^P497H^ after TNF-α treatment (Fig. 1a, b). Non-phosphorylated nuclear p65 NF-κB was equally increased in both hUBQLN2^WT^ and hUBQLN2^P497H^. We did not observe difference in nuclear phospho-p65 without TNF-α treatment but we observed an increase in cytoplasmic phospho-p65 in both hUBQLN2^WT^ and hUBQLN2^P497H^ transfected cells. We also monitored IκB-α phosphorylation and degradation as a marker of NF-κB activation. We did not observe differences in levels of phospho-IκB-α between control cells and hUBQLN2-overexpressing cells with or without TNF-α treatment. In the classical pathway of NF-κB activation, the phosphorylation of the IκB-α protein results in its ubiquitination, dissociation from NF-κB, and eventual degradation by the proteasome. However, it has been reported that in Neuro2A cells treated with TNF-*α*, IκB-α is phosphorylated and degraded within a short time period (30 min) and then the levels come back to basal level within 2 h, even if NF-κB remains activated [18]. Our results are consistent with this report. Moreover, an involvement of p38 MAPK in the phosphorylation and activation of p65 as substantiated below does not require IκB-α protein degradation.

To accurately measured the NF-κB activation, we performed a luciferase reporter assay in Neuro2A cells stably transfected with pGL4.32 [luc2p/NF-κB-RE/Hygro]. This plasmid contains a NF-κB response element which expresses luciferase when NF-κB is activated. 24 h after transfection and after 4 h of TNF-α (20 ng/ml) treatment, both hUBQLN2^WT^ and hUBQLN2^P497H^ showed a mean increase of 1.27 fold in luciferase activity (p < 0.0001) (Fig. 1c). 48 h after transfection and after 4 h of TNF-α (20 ng/ml) treatment, again both hUBQLN2^WT^ (*p* = 0.0004) and hUBQLN2^P497H^ (*p =* 0.0061) expressing cells exhibited a mean increase of 1.28 fold in luciferase activity compare to control cells (Fig. 1d).

We used siRNA to validate the role of hUBQLN2 in NF-κB activation. The main idea was to down-regulate NF-κB activity by eliminating hUBQLN2 overexpression and aggregation. Three different siRNA, purchased from Origene (Rockville), were targeting human UBQLN2 gene. All three siRNA were efficient in down-regulating the levels of hUBQLN2 in Neuro2A cells and in abrogating formation of inclusions (data not shown). We transfected Neuro2A cells with siRNA SR309321A for 48 h. 48 h after transfection, cells were treated with TNF-α for 4 h and luciferase activity was measured. Again, we detected an increase of NF-κB activity in both hUBQLN2^WT^ (1.26 fold, *p* < 0.01) and hUBQLN2^P497H^ (1.22 fold, *p* < 0.01) transfected Neuro2A cells as compared to the control cells transfected with scrambled siRNA. When hUBQLN2^WT^ or hUBQLN2^P497H^ were co-transfected with siRNA SR309321A, luciferase activity decreased (p < 0.01) and came back to level noticed in control plasmid transfected cells (Fig. 1e). Scrambled siRNA do not recognize any sequences in either mouse or human genome. We measured human UBQLN2 level after siRNA/UBQLN2 co-transfection to assure the down regulation of hUBQLN2 (Fig. 1f). Endogenous mouse UBQLN2 levels were not reduced. These results suggest a role for hUBQLN2 in modulating NF-κB activation.

### UBQLN2 expression in Neuro2A cells leads to cellular stress via MAP kinase pathway

We further investigated the cellular mechanisms which might explain NF-κB activation by UBQLN2. NF-κB can be activated by many different pathways [11]. Because most of these pathways are induced by membrane receptors, like Interleukine-1(Il-1), Toll-like receptor (TLR), tumor-necrosis factor receptor (TNFR) and growth factor receptor (GF-R), we examined intracellular pathways activated in context of cellular stress. Additionally, in Fig. 1, Neuro2A cells required to be stimulated by TNF-α to detect a significant NF-κB activation induced by UBQLN2 up-regulation. These results suggest an involvement of TNF-α downstream pathway. We hypothesized that UBQLN2 up-regulation might constitute a cellular stress which may increases levels of MAP kinases and enhances their activation by TNF-α. TNF-α receptor cascade activates numerous of signaling pathways, including p38 MAPK, p42/44 MAPK (ERK) and JNK/SAPK [19]. Each of these pathways can be activated by other mechanisms. JNK and p38 are mainly activated by cellular stress, such as inflammatory cytokines, UV, radiation, protein synthesis inhibitor, oxidative stress or ER-stress [20, 21] . ERK is more activated by growth factor, reactive oxygen species (ROS), ER-stress [22] and is also implicated in differentiation, memory and synaptic plasticity [23, 24]. All these kinases are implicated in NF-κB activation, making them suitable candidate for NF-κB activation by UBQLN2 [19].

**Fig. 2:**
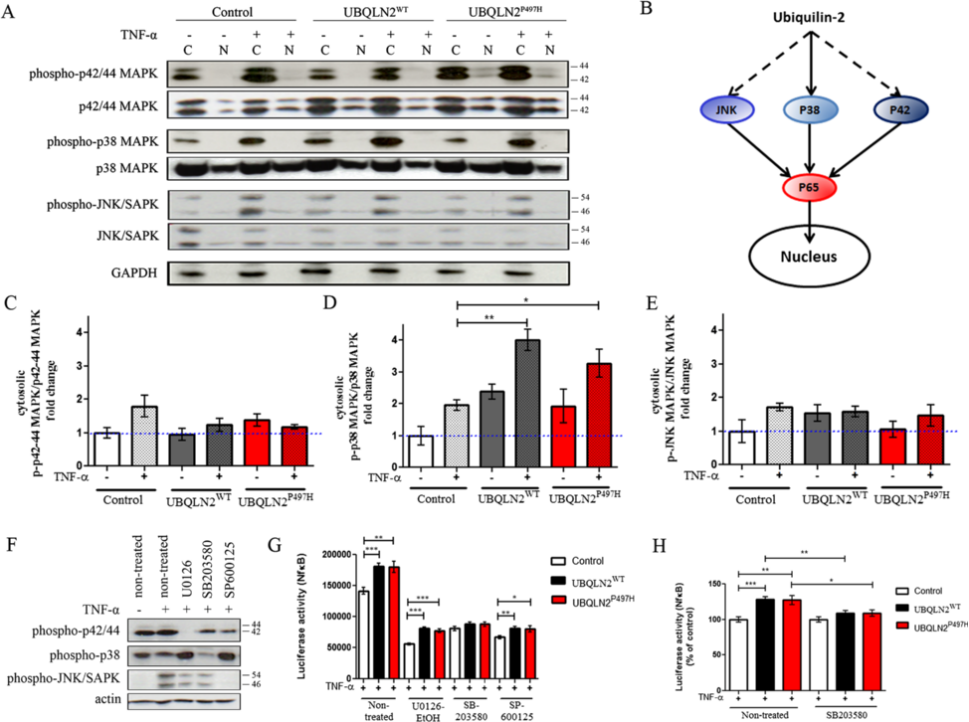
hUBQLN2 expression in Neuro2A cells caused cellular stress via MAP kinase pathway. **a** Cytoplasmic (C) and nuclear (N) extraction was realized 48 h after transfection. Neuro2A cells were treated with TNF-α 20 ng/ml for 4 h. **b** Hypothetical model of NF-κB activation by hUBQLN2. p38 MAPK seems to be a major player in this activation. **c** Quantification of cytosolic phospho-p42/44 MAPK vs non-phosphorylated p42/44 MAPK (*p* = phospho)(*n* = 3), (**d**) cytosolic phospho-p38 vs non-phosphorylated p38 MAPK (p = 0.0051 for hUBQLN2^WT^ and p < 0.05 for hUBQLN2^P497H^, *n* = 3) and (**e**) cytosolic phospho-JNK vs non-phosphorylated JNK MAPK (*n* = 3) as compared to cytoplasmic level in control cells. **f** Neuro2A cells were pre-treated 1 h with MAPK inhibitors 10 μM U0126-EtOH, SB203580, SP600125 and then treated with TNF-α 20 ng/ml for 4 h. Total protein extract was used for analysis. **g** Luciferase assay showing control plasmid, pCMV-hUBQLN2^WT^ and pCMV-hUBQLN2^P497H^ transfected Neuro2A cells. Cells were pre-treated 1 h with MAPK inhibitors 10 μM U0126-EtOH, SB203580, SP600125 and then treated with TNF-α 20 ng/ml for 4 h (*n* = 3). **h** Previous (**g**) luciferase activity was calculated in percentage of control Neuro2A cells, treated with SB203580 or not

To investigate if any of these pathways were activated by hUBQLN2, we transfected Neuro2A cells with control plasmid, pCMV-hUBQLN2^WT^ and pCMV-hUBQLN2^P497H^. We detected an increase in levels of phosphorylated MAPK in hUBQLN2 overexpressing cells. Figure 2a is showing the levels of these phosphorylated proteins (activated form) 48 h after transfection. The levels of phospho-p42/44 MAPK were not different in cells expressing hUBQLN2^WT^ and hUBQLN2^P497H^ as compared to control cells after TNF-α treatment (Fig. 2c). Moreover, there was a clear and significant increase of phospho-p38 in hUBQLN2^WT^ (p = 0.0051) and hUBQLN2P497H (p < 0.05) transfected cells treated with TNF-α (Fig. 2d). However, we did not observed any increase in phospho-JNK/SAPK level compared to control (Fig. 2e). From these results, we can propose that UBQLN2 up-regulation leads to cellular stress via MAPK pathway.

To further elucidate which MAPK pathway is implicated in NF-κB activation by UBQLN2, we treated the cells with different specific MAPK inhibitors. Luciferase activity was measured at 1 h after inhibitor pre-treatment and after 4 h of TNF-α treatment. We used U0126-EtOH, a selective p42/44 MAPK inhibitor, SB203580, a p38 MAPK inhibitor and SP600125, a selective JNK1/2/3 inhibitor. In comparison with U0126-EtOH which was specific to p42/44 MAPK inhibition, SP600125 and SB203580 also partially inhibited p42/44 MAPK at a lower level than U0126-EtOH (Fig. 2f). We observed that all three kinase inhibitors led to partial decrease of NF-κB activity by the inhibition of their specific MAPK (Fig. 2f-g). After treatment with U0126-EtOH, we determined that NF-κB activity, as compared to control cells, was still significantly increased in hUBQLN2^WT^ (1.46 fold, *p* < 0.0001) and hUBQLN2^P497H^ (1.38 fold, *p* < 0.0001) transfected cells (Fig. 2g). Consequently, treating the cells with U0126-EtOH failed to inhibit UBQLN2-mediated NF-κB activation observed with TNF-α treatment alone (Figs. 1, 2g). We obtained almost the same results with SP600125 treatment. When Neuro2A cells were treated with this JNK inhibitor, there was still an increase of NF-κB activity in cells transfected with hUBQLN2^WT^ (1.21 fold, p = 0.0034) or with hUBQLN2^P497H^ (1.20 fold *p* = 0.0301) as compared to control cells. However, after treatment with SB203580, the NF-κB activity became similar between control cells and cells transfected with hUBQLN2^WT^ (1.09 fold, *p* = 0.1052) or hUBQLN2^P497H^ (1.09 fold, *p* = 0.1690) (Fig. 2g). When measured using percentage of control, NF-κB activity after SB203580 treatment was reduced by 19.2 % in hUBQLN2^WT^ cells (*p* = 0.0038) and 18.7 % in hUBQLN2^P497H^ (*p* = 0.0289) (Fig. 2h). These results suggest that p38, but not p42/44 or JNK, was involved in enhancement of NF-κB activation by UBQLN2 up-regulation.

### UBQLN2 promotes aggregation of ALS-linked proteins and cytoplasmic mislocalization of TDP-43

**Fig. 3:**
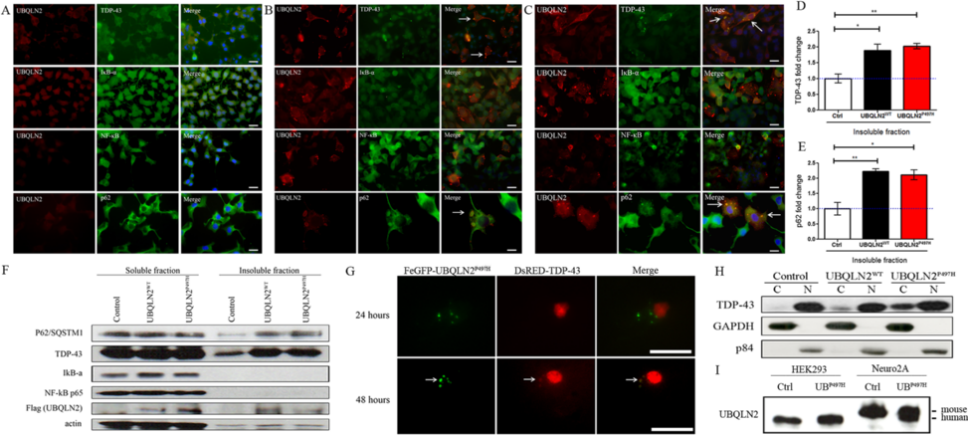
hUBQLN2 co-localized with TDP-43 and P62 but not with NF-κB and IκB-α. Immunofluorescence of Neuro2A cells at 48 h after transfection with (**a**) control plasmid, (**b**) pCMV-hUBQLN2^WT^ and (**c**) pCMV-hUBQLN2^P497H^. Indicated antibodies were used according to Materials and Methods. Microscopy pictures were taken at 63× magnification. **d**-**e** Western analysis from three repeated experiments was used for quantification of (**d**) TDP-43 level (*p* = 0.0227 for hUBQLN2^WT^ and *p* = 0.0032 for hUBQLN2^P497H^) and (**e**) p62 level (*p* = 0.0058 for hUBQLN2^WT^ and *p* = 0.0130 for hUBQLN2^P497H^) in insoluble fraction. Results were calculated in fold change compared to proteins level in insoluble fraction of control cells. **f** Neuro2A cells were transfected with control plasmid, pCMV-hUBQLN2^WT^ or pCMV-hUBQLN2^P497H^. Cells were extracted 48 h after transfection using aggregates assay protocol. The results suggested that overexpression of UBQLN2 species increased levels of UBQLN2, TDP-43 and p62 in the insoluble fraction. **g** Live imaging was performed on Neuro2A pFeGFP-hUBQLN2^P497H^ and DsRED-TDP-43 co-transfected cells. Microscopy pictures were taken at 24 and 48 h after transfection. **h** Immunoblot of TDP-43 in cytoplasmic (C) vs nuclear (N) after transfection with control plasmid, pCMV-hUBQLN2^WT^ or pCMV-hUBQLN2^P497H^ plasmids. **i** HEK293 cells and Neuro2A cells were transfected with either control plasmid or pCMV-hUBQLN2^P497H^ plasmid. Protein levels of hUBQLN2 and mUBQLN2 were measured at 48 h after transfection. Human UBQLN2 levels were similar in control HEK293 and pCMV-hUBQLN2^P497H^ Neuro2A transfected cells. Scale bar = 25 μm

We investigated a potential involvement of protein aggregation in cellular stress due to UBQLN2 expression. Protein inclusions are a pathological hallmark of ALS and other neurological disorders. Proteins such as TDP-43, SOD1, P62 and FUS are components of these aggregates [25]. It has been shown with Neuro2A cells that co-transfection of UBQLN2 and c-terminal domain of TDP-43 cause aggregates of both proteins and that UBQLN2 seem to be more prone to aggregation than TDP-43 [4]. Furthermore, UBQLN2^P506T^ co-localize with TDP-43 when injected in mouse with rAAV2/8 [26], but not in UBQLN2^P497H^ transgenic mouse [27] or in UBQLN2^P497H^ transgenic rats [28]. To elucidate this inconstancy and to clarify if hUBQLN2 aggregates can promote aggregation of endogenous TDP-43, we used different biochemical methods. First, we transfected Neuro2A cells with pCMV-hUBQLN2^WT^ or pCMV-hUBQLN2^P497H^ to determine if inclusions were formed and if they exhibited ALS-like features with aggregated TDP-43 or p62 proteins and if positive for NF-κB associated proteins. Immunofluorescence microscopy revealed co-localization of UBQLN2, TDP-43 and p62 (as expected), but not co-localization of NF-κB p65 or IκB-α (Fig. 3b-c). Similar results were obtained when transfected cells were treated with TNF-α (data not shown). These data suggest the lack of direct interaction between UBQLN2 and NF-κB or IκB-α.

UBQLN2 aggregation triggered a mislocalization of endogenous TDP-43 from nucleus to cytoplasm. Analyses of cytoplasmic and nuclear extracts revealed an increase in cytoplasmic TDP-43 in both hUBQLN2^WT^ and hUBQLN2^P497H^ transfected cells compare to control, at 48 h after transfection (Fig. 3h). To further confirm the formation of aggregates, we have fractionated the soluble and insoluble proteins. There was significant increase of TDP-43 and p62 levels in insoluble fraction of hUBQLN2-expressing cells as compared to control cells (Fig. 3d, e, f). There was no increase of NF-κB or IκB-α in the insoluble fraction of UBQLN2 cells. To examine if the cytosolic mislocalization of TDP-43 by UBQLN2 is time dependent, we performed live imaging of Neuro2A cells co-transfected with pFeGFP-hUBQLN2^P497H^ and DsRED-TDP-43. We noted that at 24 h after transfection, TDP-43 was localized in the nucleus whereas UBQLN2^P497H^ was detected in cytoplasmic inclusions. However, at 48 h after transfection, TDP-43 was co-localized with UBQLN2-positive inclusions (Fig. 3g). To assess whether the expression levels of hUBQLN2 in transfected Neuro2A were of physiological relevance, we compared the levels of UBQLN2 in transfected and non-transfected Neuro2A cells with UBQLN2 levels in non-transfected and transfected HEK293 cells. The immunoblotting results revealed that levels of hUBQLN2 in transfected Neuro2A cells were not in excess of endogenous mouse ubiquilin-2 levels or of endogenous human UBQLN2 levels in HEK293 cells (Fig. 3i). So, this suggests that small changes in UBQLN2 levels are sufficient to promote cytosolic co-aggregation of UBQLN2, TDP-43 and p62. It should be noted that we did not detect TDP-43 aggregates in Neuro2A cell overexpressing only CMV-TDP-43 vector (Additional file 1).

### UBQLN2 aggregates are dynamic structures

**Fig. 4:**
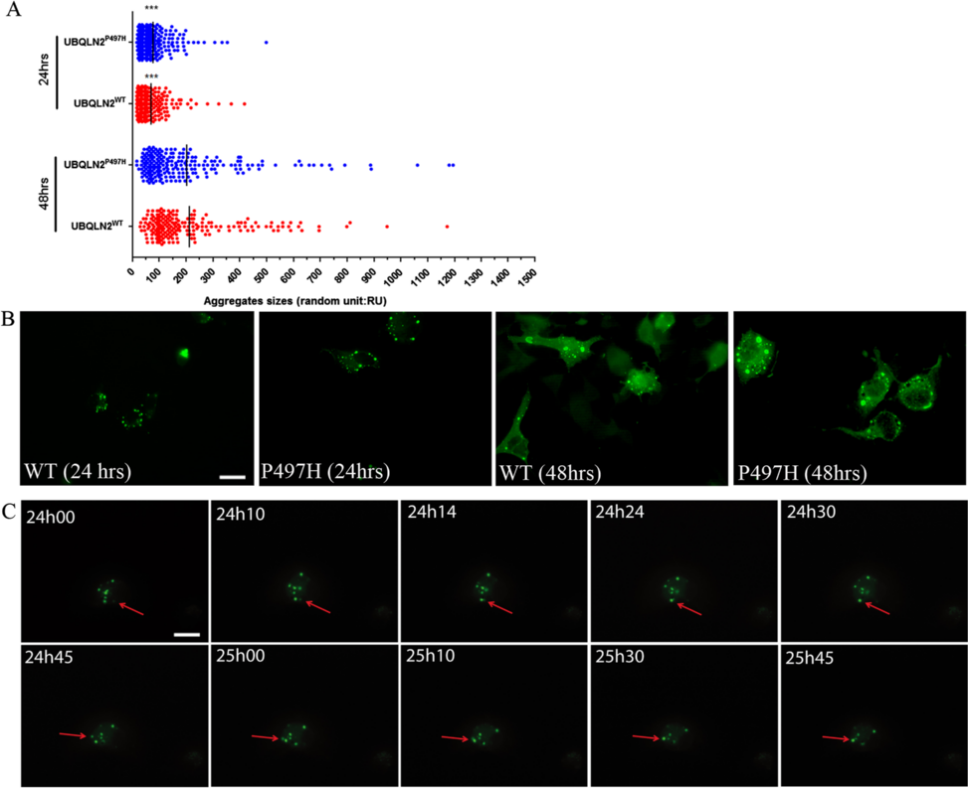
hUBQLN2 inclusions were dynamic. **a** Immunofluorescence of GFP-labeled hUBQLN2 proteins at 24 h and 48 h after transfection of Neuro2A cells with pFeGFP-hUBQLN2^WT^ or pFeGFP-hUBQLN2^P497H^ was used to measure the size of inclusions (>200 aggregates, *p* < 0.0001). Fifty cells per group were examined. **b** Immunofluorescence showing representative pictures at each time point (magnification at 63×). **c** Live imaging was done on Neuro2A pFeGFP-hUBQLN2^P497H^ transfected cells. Pictures were taken each minute over a 2 h period starting at 24 h post-transfection. Red arrow is showing moving aggregates (Additional file 2: Move S1). Scale bar = 25 μm

To visualize the formation of protein inclusions, we carried out a time-lapse microscopy imaging of Neuro2A cells transfected with pFeGFP-hUBQLN2^WT^ or pFeGFP-hUBQLN2^P497H^. A two hours imaging study carried out at 24 h after transfection revealed the dynamic nature of aggregates in hUBQLN2 transfected cells. Figure 4c shows merging aggregates over a 2 h period of time in pFeGFP-hUBQLN2^P497H^ cells. We observed that smaller inclusions combined with bigger one (Additional file 2: Movie S1). To further investigate this phenomenon, we took pictures of fifty FeGFP-hUBQLN2 positive cells at 24 and 48 h and measured each aggregates size. The mean aggregates sizes were 69.22 ± 4.032 RU in UBQLN2^WT^ at 24 h and 213.9 ± 12.99 RU at 48 h after transfection (Fig. 4a, b). Similar results were obtained with hUBQLN2^P497H^ (77.03 ± 3.70 and 203.1 ± 13.87 at 24 and 48 h, respectively). We measured over 200 aggregates in each group and did not see significant difference in the number of aggregates per cell. The size difference between 24 h and 48 h was significant (*p* < 0.0001). We were also able to observe formation of aggregates after few hours of transfection, confirming that hUBQLN2 is prone to aggregation [4]. We also measured aggregates size when transfected with pCMV-hUBQLN2 (without GFP tag) and similar results were obtained. Aggregates merged and became bigger over time after transfection. These results suggest that, like stress granules, hUBQLN2 inclusions are dynamic in neurons and they can sequester TDP-43 in the cytosol.

### UBQLN2 up-regulation enhances vulnerability to NF-κB-mediated neuronal death and causes an ER-stress

**Fig. 5:**
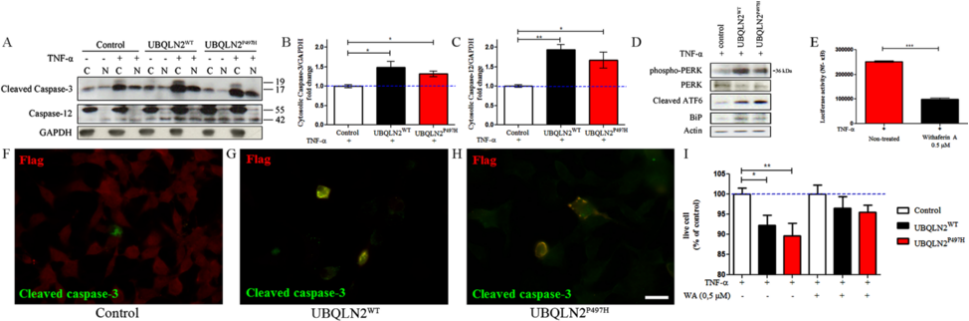
UBQLN2 induced a NF-κB-mediated neuronal cell death and an ER-stress response. **a** Neuro2A cells were transfected with control plasmid, pCMV-hUBQLN2^WT^ or pCMV-hUBQLN2^P497H^ and collected after 48 h including 4 h TNF-α 20 ng/ml treatment, (C) cytoplasmic and (N) nuclear. Quantification of (**b**) cleaved-caspase-3 (*n* = 3) and (**c**) caspase-12 (n = 3) vs GAPDH as compared to cytoplasmic level in control cells when treated with TNF-α. (**d**) Total proteins extract was used for western analysis of ER-stress marker 48 h after transfection of control plasmid pCMV-UBQLN2^WT^ and pCMV-UBQLN2^P497H^. (**e**) Neuro2A cells were stably transfected with pluc2p-NFκB-RE plasmid and then treated with Withaferin A (WA) 0.5 μM for 2 h previous to TNF-α 20 ng/ml treatment. Luciferase activity (NF-κB) showed a significant decreased in NF-κB activity after WA treatment (*n* = 3, *p* < 0.0001). (**f**-**h**) Immunofluorescence of Neuro2A-transfected cells fixed in PFA 4 % at 48 h and then labeled with cleaved caspase-3 (green) and flag (UBQLN2) (red) in (**e**) control transfected cells (**f**) hUBQLN2^WT^-transfected cells and (**g**) hUBQLN2^P497H^-transfected cells. Scale bar = 25 μm. (**i**) MTS assay was realized on cells at 48 h after transfection. The number of live cells was represented using percentage of live cell as compared to control transfected cells. We observed significant mortality in hUBQLN2^WT^ (*p* = 0.0144, n = 3) and hUBQLN2^P497H^ (*p* = 0.0067, n = 3) transfected cells. Cells were also treated with Withaferin A 0.5 μM for 2 h previous to TNF-α 20 ng/ml treatment

To further examine whether aggregates of both hUBQLN2^WT^ and hUBQLN2^P497H^ are associated with cellular stress with ensuing neuronal death, we monitored cell survival and apoptosis after UBQLN2 transfection. In ALS spinal cord, there is evidence of inverse correlation between ubiquitinated inclusions and the number of motor neurons, suggesting a link between aggregates and cell toxicity [29]. Moreover, NF-κB activation induced by TNF-α was found to induce motor neuron death in context of glutamate excitotoxicity [30]. Neuro2A cells were transfected with control plasmid vector, pCMV-hUBQLN2^WT^ and pCMV-hUBQLN2^P497H^ vectors. We then examined the activation of caspase-3, a marker of apoptosis. We observed that cleaved-Caspase-3 was increased in UBQLN2^WT^ (p = 0.039) and UBQLN2^P497H^ (p = 0.0167) transfected and TNF-α-treated cells after 48 h compared with control cells (Fig. 5a, b). Immunofluorescence microscopy showed that UBQLN2 positive cells were also positive for caspase-3 (Fig. 5f-h). We performed a MTS assay in transfected cells at 48 h. The number of viable cells were decreased in both UBQLN2^WT^ (p = 0.0144) and UBQLN2^P497H^ (*p* = 0.0067) transfected cells (Fig. 5i).

To assess the contribution of NF-κB activation to neuronal death of UBQLN2 overexpressed cells, we treated the cells with Withaferin A (WA), a known NF-κB inhibitor [31]. Neuro2A cells were pre-treated with WA 0.5 μM for 2 h previous to TNF-α 20 ng/ml treatment. First, we observed in luciferase assay of non-transfected cells that WA decreased NF-κB activity by 2.5 times (p < 0.0001) (Fig. 5e). Again, we performed a MTS assay in transfected and WA treated cells and observed that Withaferin A succeeded to decreased mortality previously found in Neuro2A transfected cells (Fig. 5i). These results suggested that UBQLN2 enhanced vulnerability to neuronal death caused by TNF-α-mediated NF-κB activation.

We also looked at upstream caspase activation to clarify cellular mechanisms under this apoptosis induction. Cleaved-caspase-12, responsible for ER-stress-induced apoptosis, was increased in hUBQLN2^WT^ (p = 0.0023) and hUBQLN2^P497H^ (p = 0.0309) transfected cells (Fig. 5a and c). It has been shown that UBQLN2 mutation leads to proteasome impairment [4] and impairs endoplasmic reticulum-associated protein degradation (ERAD) [32]. Normally, ER proteins which fail to fold correctly are degraded by the 26S proteasome but when proteasome is impaired, ER stress is induced by the accumulation of ERAD substrates [33]. To confirm if the caspase-12 increase can be explain by UBQLN2-mediated ER-stress, we quantified ER-stress markers.

Protein aggregates causing ER-stress leads to increased levels of Binding immunoglobulin protein (BiP), an ER chaperone. In circumstance of accumulation of unfolded proteins in the ER, BiP induces activation of different transmembrane proteins like protein kinase RNA-like endoplasmic reticulum kinase (PERK) or Activating transcription factor 6 (ATF6). BiP levels were increased in hUBQLN2^WT^ and in hUBQLN2^P497H^ transfected Neuro2A (Fig. 5d). ATF6 is a membrane-anchored key transcription factor of the unfolded protein response (UPR) [34]. We observed an increase in cleaved fragment of ATF6 (36 kDa) in both hUBQLN2^WT^ and hUBQLN2^P497H^. Phospho-PERK was also increased in UBQLN2 transfected cells. From these results, we can postulate that UBQLN2 aggregates (unfolded proteins) and ERAD degradation impairment [32] lead to BiP activation and BiP induced an ER-stress response.

## Discussion

Here, we report that an upregulation of Ubiquilin-2 species can enhance NF-κB activity in neuronal cells. Many lines of evidence support this conclusion: (a) nuclear levels of NF-κB phospho-P65 were increased in Neuro2A cells overexpressing hUBQLN2^WT^ and hUBQLN2^P497H^, and after TNF-α treatment (Fig. 1a, b); (b) luciferase reporter activity of NF-κB was also increased in cells overexpressing hUBQLN2^WT^ or hUBQLN2^P497H^ (Fig. 1c, d); and (c) down-regulation of hUBQLN2 by the use of siRNA targeting hUBQLN2 succeeded in reducing NF-κB activity to its basal level (Fig. 1e). So, we detected significant increase of NF-κB activity by UBQLN2 species up-regulation using three different approaches. It should be noted that an up-regulation of UBQLN2 alone was not sufficient to activate NF-κB and that Neuro2A cells required to be primed with TNF-α.

These results are consistent with the view of a converging role for NF-κB in ALS pathogenesis. Several ALS-linked proteins were found to modulate the NF-κB pathway: TDP-43 upregulation can enhance NF-κB activation [14], mutation in valosin-containing protein (VCP) in mice resulted in NF-κB hyperactivation [35, 36] and suppression of Optineurin (OPTN) led to neuronal death via NF-κB pathway [37]. Fuse in Sarcoma (FUS) was found to enhance NF-κB activation induced by physiological stress [38] and NF-κB-related inflammation was increased in mouse deficient in Progranulin (PGRN) [39]. Furthermore, there are reports that NF-κB inhibition by Withaferin A conferred neuroprotection in transgenic mouse models [14, 15].

Our findings that an up-regulation of UBQLN2 species enhances NF-κB activity in a TNF-α treatment dependent manner (Fig. 1) led us to investigate the cellular mechanisms underlying this activation. Evidence for MAPK implication came from the observation that: (a) phospho-p38 MAPK levels were increased in cells transfected with UBQLN2 species mainly with TNF-α treatment (Fig. 2a and d) and (b) there was inhibition of NF-κB activation due to UBQLN2 species by treatment with p38 inhibitor but not with p42/44 or JNK inhibitors (Fig. 2g, h). Thus, our results suggest an involvement of p38 MAPK in the enhancement of NF-κB activation by UBQLN2. Figure 2b is showing a hypothetical model of NF-κB activation by UBQLN2 inclusions. Because phospho-p38 MAPK was robustly increased in UBQLN2 overexpressing cells and because its inhibitor SB203580 blocked a significant increase of NF-κB activity in UBQLN2 species-transfected Neuro2A cells, we propose that p38 is the main activator of NF-κB p65 due to UBQLN2 overexpression. This is the first report of NF-κB activation via p38 MAPK pathway by an up-regulation of ALS-linked UBQLN2 mutant. This finding is in line with previous reports of up-regulation of p38 MAPK levels in motor neurons of SOD1^G93A^ mice [40, 41].

A common pathological feature of ALS/FTD is the redistribution of TDP-43 from nucleus to cytoplasm in neurons of spinal cord and brain [42]. Here, we report that physiological (Fig. 3i) expression of hUBQLN2^WT^ or hUBQLN2^P497H^ in Neuro2A can promote formation of cytoplasmic inclusions that progressively sequester TDP-43. The evidence is based on the immunolocalization of UBQLN2 and TDP-43 (Fig. 3a, c), the increased TDP-43 levels in insoluble fraction of Neuro2A transfected with UBQLN2^WT^ and UBQLN2^P497H^ (Fig. 3d, e, f) and the increased levels of TDP-43 in the cytosol of Neuro2A cells overexpressing UBQLN2 species (Fig. 3h). These results are consistent with previous reports that ALS patients with UBQLN2 mutation are exhibiting UBQLN2 aggregates positives for TDP-43 [4, 5] and that UBQLN2 binds TDP-43 with high affinity [43]. A co-localization of either UBQLN2^WT^ or UBQLN2^P497H^ with c-TDP-43 has also been shown in Neuro2A cells co-transfected with c-TDP-43 and UBQLN2 [4] but not in UBQLN2^P497H^ transgenic mouse [27] or in UBQLN2^P497H^ transgenic rats [28]. This inconsistency can be explained by the low to moderate level of UBQLN2 protein in these in vivo models. It has been proposed that interaction and aggregation between UBQLN2 and TDP-43 is concentration dependent [4, 43]. Indeed, we noted that TDP-43 was easier to detect in UBQLN2 aggregates at 48 h than 24 h after transfection which correlate to UBQLN2 aggregates sizes (Fig. 3g-4a).

So, factors in ALS which may contribute to cytosolic accumulation of UBQLN2 such as proteasome deficiency [4, 44] might also contribute to cytosolic TDP-43 accumulations. The early pathogenic mechanisms underlying TDP-43 recruitment to UBQLN2 inclusions has to be clarified. Recent report suggested that the binding of UBQLN2 to the C-terminal tail of TDP-43 reduces TDP-43 affinity for nucleic acids and may inhibit its physiologic function and increase its aggregation [43]. Another study has suggested that the oxidation of RRM1 domain of TDP-43 may cause protein aggregation [45]. Although, there is substantial evidence for oxidative stress in ALS physiopathology [46], it is still unknown whether UBQLN2 aggregation can cause oxidative stress which lead to TDP-43 RRM1 domain oxidation. However, we report that UBQLN2 up-regulation cause a cellular stress which leads to MAPK activation. Interestingly, kinases have been recently shown to have a critical role in TDP-43 accumulation in stress granules following a chronic stress [47]. The phosphorylation by kinases may modulate the association of TDP-43 with stress granules and, through a chronic process, could lead to the pathogenic aggregates find in ALS. Consequently, a time dependent recruitment of TDP-43 in UBQLN2 inclusions (Fig. 3g) could be explained in part by chronic kinases activation caused by UBQLN2 dysregulation.

We propose that UBQLN2 overexpression increased vulnerability to neuronal death. Many lines of evidence support this conclusion: (a) protein levels of cleaved caspase-3 and caspase-12, an ER-stress related caspase, were increased in cells overexpressing UBQLN2 species when treated with TNF-α (Fig. 5a); (b) most of cleaved caspase-3 positive cells were positive for UBQLN2 (Fig. 5f, h); and (c) the number of living cells counted by MTS assay were decreased in Neuro2A transfected with UBQLN2^WT^ or UBQLN2^P497H^ (Fig. 5i). Moreover, we observed that UBQLN2-induced neuronal death can be decreased with WA treatment (Fig. 5i), which could suggest a role for NF-κB activation in neuronal death. We have previously shown that TDP-43 overexpression can enhance microglial toxicity toward neighboring neurons via NF-κB pathway and that NF-κB inhibition by Withaferin A treatment of TDP-43 mouse model reduces ALS disease symptoms [14]. Withaferin A also reduced levels of misfolded SOD1 and extended lifespan of mutant SOD1 ALS mice [15]. NF-κB is also known to modulates apoptosis in neurons treated with glutamate [48] or with chemicals inducing DNA-damage [49]. Our results are similarly consistent with previous studies with UBQLN2^P497H^ rats reporting neuronal loss in the cortex and dentate gyrus and a glial activation surrounding neuronal damages [28]. These results, taken together, suggest that the up-regulation of UBQLN2 species can act like TDP-43 toxicity in neurons and that neurons bearing UBQLN2 inclusions become more vulnerable to toxic mediator TNF-α secreted by activated microglia in context of inflammation.

There is evidence that mutations in UBQLN2 can slow down degradation of this protein [4, 44]. Thus, such impairment of protein turnover can lead to an increase in the steady state levels of mutant UBQLN2. A similar post-transcriptional dysregulation in levels of WT UBQLN2 in ALS cases without genetic mutation cannot be excluded. Indeed, UBQLN2 accumulations have been detected in ALS patients without a mutation in the ubiquilin-2 gene [4]. Our observation that both WT and mutant UBQLN2 species can form inclusions when overexpressed in transfected Neuro2A cells (Fig. 3) are in line with data previously reported by Deng et al. [4].

## Conclusion

In conclusion, our results suggest that dysregulation by up-regulation of UBQLN2 may contribute in part to sporadic and familial ALS pathogenesis through enhancement of NF-κB activation by p38 MAPK signaling and formation of inclusion bodies sequestering TDP-43. These pathways might represent suitable therapeutic targets for future ALS treatment.

## Methods

### Cell culture, transfection and cell treatment

Almost all experiments were done with Neuro2A cells, which are mouse neuroblastoma cells. Neuro2A cells were growth in Dulbecco’s Modified Eagle Medium (DMEM) with 10 % fetal bovine serum (FBS), 1 % penicillin-streptomycin and 1 % glutamine. Cells were transfected at 80-90 % confluence with lipofectamine 2000 according to manufacturer protocol. Opti-MEM media was replaced by normal growth media 24 h after transfection. PCMV-hUBQLN2^WT^ and pCMV-hUBQLN2^P497H^ were used for transfection (see section plasmids construction). We used pCDNA3 as control plasmid. Cells were collected at 24 h or 48 h after transfection consistent with the experiment. Recombinant mTNF-α (R&D systems, Minneapolis) treatment (20 ng/ml) was done 4 h before collecting the cells. HEK293 cells, which are human embryonic kidney cells, for Fig. 3i were cultured with same protocol as Neuro2A.

For kinase pathways investigation, MAPK specific inhibitors U0126-EtOH, SP600125 and SB203580 (ApexBio, Houston) dissolved at 1 mg/ml in DMSO were used at 10 μM in a 1 h pre-treatment and then TNF-α (20 ng/ml) was added to the cells for 4 h. Human UBQLN2 siRNAs were purchased from Origene (catalog No: SR309321, Rockville). SiRNA SR309321A: rGrGrCrArGrCrUrCrArUrUrArUrGrG rCrUrArArUrCrCrACA was used for the experiments. Cells were co-transfected with siRNA (10 nM) and control plasmid, pCMV-hUBQLN2^WT^ or pCMV-hUBQLN2^P497H^. NF-κB activation (luciferase assay) was measure at 48 h after transfection and after 4 h TNF-α treatment.

### Protein extraction and Western blot analysis

After collecting the cells, cytoplasmic and nuclear extraction or total proteins extraction was realized. Cytoplasmic buffer contained 10 mM HEPES pH 7.5, 10 mM KCl, 1.5 mM MgCl_2_, 0.34 M sucrose, 10 % glycerol, 1 mM PMSF, 10 mM NaF and 1 mM Na_2_VO_3_. After ice lysis and low speed centrifugation, nuclear fraction was obtained with sonication in buffer containing 3 mM EDTA, 0.2 mM EGTA, 1 mM PMSF, 10 mM NaF, 1 mM Na_2_VO_3_. 30 μg of proteins was loaded into 10 % SDS-page gels. Total protein was obtained with buffer containing 0.15 M NaCl, 0.05 M tris pH 7.4, 10 % glycerol and 1 % Triton X-100. Cells were incubated on ice for 30 min and then centrifuged to collect the supernatant.

For aggregates assay, we first collected the cells at 48 h after transfection and dissolved them into a re-suspending buffer containing 10 mM tris, 1 mM EDTA and 100 mM NaCl. Then, we added same volume of extraction buffer 1 containing 10 mM Tris, 1 mM EDTA, 100 mM NaCl, 1 % NP-40, protease and phosphatase inhibitor 1X. After sonication and high speed centrifugation (>100 000 g) for 5 min, we kept the supernatant (soluble fraction). We added extraction buffer 2 containing 10 mM Tris, 1 mM EDTA, 100 mM NaCl, 0,5 % NP-40 and protease and phosphatase inhibitor 1X to the pellet 1, sonicated and centrifuged again (>100 000 g for 5 min). After removing the supernatant, we extracted the pellet 2 with 10 mM Tris, 1 mM EDTA, 100 mM NaCl, 0,5 % NP-40, 0,25 % SDS, 0,5 % Deoxycholic acid and phosphatase and protease inhibitor 1X. Finally, we sonicated and kept supernatant 3 (insoluble fraction) [50].

Antibodies used were phospho-NF-κB p65 (Cell signaling, Whitby, 1:1000), NF-κB p65(Santa-cruz, Dallas, 1:1000), phospho-IκB-α (Cell signaling, 1:1000), IκB-α (Santa-Cruz, 1:1000), actin (Milipore, Etobicoke, 1:20,000), p84 nuclear matrix (Abcam, Cambridge, 1:1000), GAPDH (Abcam, 1:1000), TARDBP (Proteintech, Chicago, 1:2500), FLAG M2 (Sigma-Aldrich, Saint-Louis, 1:1000), UBQLN2 (Abcam, 1:1000), phospho-SAPK/JNK (Cell signaling, 1:1000), SAPK/JNK (Cell signaling, 1:1000), phospho-p42/44 (Cell signaling, 1:1000), p42/44 (Cell signaling, 1:1000), phospho-p38 (Cell signaling, 1:1000) p38 (Cell signaling, 1:1000), cleaved-caspase-3 (Cell signaling, 1:1000), caspase-12 (Cell signaling, 1:1000), phospho-PERK (Cell signaling, 1:1000), PERK (Sigma-Aldrich, 1:1000), ATF6 (Imgenex, 1:1000) and BiP (Cell signaling, 1:1000).

### Luciferase assay

Neuro2a cells were stably transfected with pGL4.32 [luc2p/NF-κB-RE/Hygro] (Promega, Madison) and then transfected with previous plasmids. After 24 or 48 h, cells were lysed with glo lysis buffer (Promega, Madison) and 1 volume of Bright Glo luciferase assay system (Promega, Madison) was added after 5 min. Each sample was duplicated and the experiments were repeated more than 5 times. Luciferase activity was measured with Enspire reading machine in a 96 wells plate.

### Immunofluorescence

48 h after transfection with pCMV-UBQLN2^WT^ and pCMV-UBQLN2^P497H^, cells were fixed with 4 % PFA and methanol on 10 mm coverslip. Goat serum 10 % was used for blocking. First antibody was incubated overnight at 4 °C and 1 h at room temperature for secondary antibody. Primary antibodies were monoclonal Flag M2 (Sigma-Aldrich, Saint-Louis, 1:100), TARDBP (Proteintech, Chicago, 1:600), NF-κB p65 (Santa-Cruz, 1:200), IκB-α (Santa-Cruz, 1:200) and SQSTM1/P62 (Cell signaling, 1:200). Secondary antibodies were alexa-fluor 488 goat anti-rabbit (1:500) and alexa-fluor 594 goat anti-mouse (1:500).

### Plasmids construction

Human UBQLN2 gene was obtained by PCR from RP11 human BAC: 43 N15. Gene was amplified using the primer 5′GGGGAATT CATGGACTACAAGGACGACGATGACAA GGCTGAGAATGGCGAGAGCAGCGGC-3′ (forward) and 5′-GGGGCGGCCGC TGGGGT GGGATAATCCTCCTAAAC-3′ (reverse). The forward primer introduced an EcoRI restriction site and a flag tag in C-terminal. The reverse primer introduced a NotI restriction site. The P497H mutation was introduced by mutagenesis. The PCR products were ligated into pCDNA3 plasmids with same restriction sites. The plasmids finally drove human UBQLN2 wild-type or P497H mutant c-terminally fused to Flag under the control of a CMV promoter. We named these two plasmids pCMV-hUBQLN2^WT^ and pCMV-hUBQLN2^P497H^.

To create a FeGFP-hUBQLN2 plasmid, we amplified UBQLN2 sequence from previous pCMV-UBQLN2 plasmids by PCR using the primer 5′-dGGGACGACGGATCCG CTGAGAATGGCGAGAGCAGCGGCCC-3′ (forward) and 5′-dGGGACGACGCGG CCGCTTACGATGGCTGGGAGCCCAG -3′ (reverse). The forward and reverse primers introduced BamHI and NotI restriction sites without the flag tag in c-terminal. The PCR product was ligated into pCDNA3-Flag-eGFP plasmid with the same restriction sites. The plasmids finally drove human UBQLN2 c-terminally fused to Flag-eGFP under the control of a CMV promoter. We named these two plasmids pCMV-FeGFP-hUBQLN2^WT^ and pCMV-FeGFP-hUBQLN2^P497H^. Human wild type TDP-43 gene was used for DsRed-TDP-43 plasmid construction like previously described [51].

### MTS assay

The number of live cells in proliferation was measured by MTS assay 48 h after transfection. Celltiter 96 AQ_ueous_ One Solution Cell Proliferation Assay (Promega, Madison) was a colorimetric assay for determining the cell viability. MTS tetrazolium compound was reduced in a colored formazan by live cells. Cells were growth in a 24 wells plate. Live cells were washed with PBS *X*2 and then collected. We took 100 μl of media and putted them in a 96 wells reading plate. 20 μl of One solution Reagent was added and cells were incubated at 37 °C for 1 h. Absorbance was measured at 490 nm. Viable cell was calculated by dividing absorbance in transfected cells by absorbance in non-transfected cells and then reported in percentage of control transfected cells. Cells were either treated or not with Withaferin A (Enzo Life Sciences) 0.5 μM for 2 h and TNF-α 20 ng/ml was added to media for 4 h.

### Live cell imaging

Neuro2A cells were transfected with pCMV-FeGFP-hUBQLN2^WT^, pCMV-FeGFP-hUBQLN2^P497H^ or DsRed-TDP-43. After 24 h, opti-MEM was removed and replaced by DMEM 10 % FBS. Cells were then putted in NIKON ECLIPSE TE2000-E live imaging system. Pictures were taken every 2 min over a 2 h period using Metamorph software.

### Statistical analysis

Statistical significance was assessed using GraphPad Prism software. We used Student’s unpaired *t*-test for generating the p-values. We considered p < 0.05 as statistically significant.

## Additional files

Additional file 1:
**Lack of cytoplasmic inclusions in neuro2A cells overexpressing TDP-43.** Immunofluorescence of Neuro2A cells at 48 h after transfection with pCMV-TDP-43 vector only. TDP-43 is mainly expressed in nucleus. No TDP-43 aggregates were detected in cytosol of transfected cells. Scale bar = 25 μm. (JPEG 1482 kb) Additional file 2: Movie S1. Movement and fusion of UBQLN2 inclusions in Neuro2A. Live imaging was performed on Neuro2A pFeGFP-hUBQLN2^P497H^ transfected cells. Pictures of a single cell were taken each minute over a 2 h period starting at 24 h after transfection (*n* = 3). Neuro2A transfected with pFeGFP-hUBQLN2^WT^ display the same aggregates movement. We observed different aggregates combining together over this period. Small aggregates seemed to all combined with the bigger aggregate. (AVI 467717 kb)
