# Supplementary figures and images for: Ubiquilin-2 drives NF-κB activity and cytosolic TDP-43 aggregation in neuronal cells
Source: Mol Brain. 2015 Oct 31;8:71. doi: 10.1186/s13041-015-0162-6 (PMC4628361; doi:10.1186/s13041-015-0162-6)

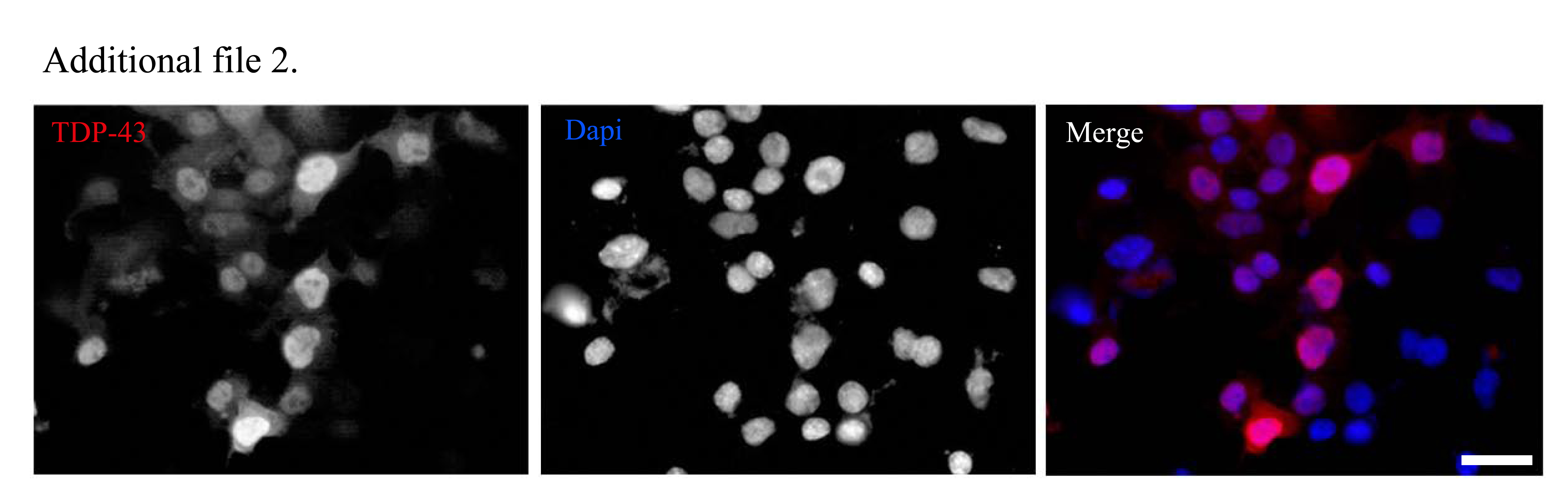

Supplement: Additional file 1: — Lack of cytoplasmic inclusions in neuro2A cells overexpressing TDP-43. Immunofluorescence of Neuro2A cells at 48 h after transfection with pCMV-TDP-43 vector only. TDP-43 is mainly expressed in nucleus. No TDP-43 aggregates were detected in cytosol of transfected cells. Scale bar = 25 μm. (JPEG 1482 kb) [file 13041_2015_162_MOESM1_ESM.jpg]
